# Tenacity

**DOI:** 10.1016/j.shj.2025.100470

**Published:** 2025-05-28

**Authors:** Anthony DeMaria

**Affiliations:** Judy and Jack White Chair in Cardiology, Sulpizio Cardiovascular Center, University of California, San Diego, California, USA

Few areas of medicine have undergone as successful, important, and impactful innovations as cardiovascular disease. The list is extensive, ranging from statins and antiplatelet agents, coronary angioplasty and stents, automated implanted cardiac defibrillators, and, of course, transcatheter aortic valve replacement. The process of innovation that led to these discoveries has been a wonder that typically involved a number of setbacks on the way to ultimate success. I have been privileged to know many of the innovators responsible for these advances and to witness presentations of their journey to final accomplishment. In virtually every presentation that I can recall, the inventors have discussed the need for tenacity and its importance to achieving their ultimate goal. Recently, I had the opportunity to hear a presentation that represented the quintessential example of the importance of persistence in the development of novel therapies.

Renal artery sympathetic denervation is now of documented efficacy for the treatment of resistant hypertension, has received approval from the Food and Drug Administration, and is reimbursed by many payors. A number of well-designed and conducted prospective, randomized, double-blinded trials have provided evidence of efficacy for renal denervation in the absence and presence of antihypertensive drug therapy and in the short- and longer-term periods.^1^^,^^2^ While careful patient selection remains paramount and the expertise of the operator is imperative, there is no longer debate that in the right patient and the right hands, the therapy can be beneficial. However, this was not always the case.

The story of the circuitous journey of renal denervation to the demonstration of efficacy and acceptance is generally well known. It is not my purpose to reexamine these events in detail. However, some general review is useful. As I discussed in another Editor’s Page over a decade ago,^3^ early observational studies and randomized trials without a sham control all yielded evidence of statistically significant blood pressure reduction in resistant hypertension patients. These data led to numerous publications in high-impact factor journals, the awarding of the European CE Mark, massive expenditure for the acquisition of the technology, and brisk clinical application in an estimated 10,000 patients. However, the first sham-controlled trial (SYMPLICITY HTN 3) yielded the somewhat shocking results that renal denervation was not significantly superior to optimal medical therapy.^4^ These unexpected results led to a virtual cessation of development, exploration, and application of the technique. It seemed to many that the question of the potential of renal denervation to be of benefit was answered, and the answer was negative.

However, as has been true of virtually every other major innovation, there were those who believed in the technology and regarded the results of SYMPLICITY HTN 3 as a mere setback. They had the tenacity to persist in the development of the therapy. Without going into detail, greater information on the anatomy of renal artery sympathetic innervation, enhancement of the instrumentation used to perform denervation, and modification of the procedure, among other advances, lead to the documentation of statistically significant benefit in sham-controlled trials. The result was the presentation that I recently attended that demonstrated the benefit of renal denervation in clinical practice.

It is not my intention to praise the virtues of renal denervation. Although I am impressed with the evidence supporting its efficacy, I have not yet had a patient who I felt should be referred for the procedure. However, I do want to extol those who persisted in developing and refining the procedure. In a sense, these individuals succeeded in bringing renal artery sympathetic denervation “back from the dead.” Time after time it is this type of tenacity that succeeds in overcoming setbacks and bringing new discoveries to clinical care. I have heard inventors say that failure is an important step in innovation since it informs and directs future work. At a time when a plethora of novel therapies for cardiovascular disease are on the drawing board, especially for structural heart disease, this is an important message to keep in mind, and a crucially important characteristic for emerging inventors.

## Funding

The author has no funding to report.

## Disclosure Statement

The author reports no conflict of interest.
